# Patients’ Perspectives on Plans Generated During Primary Care Visits and Self-Reported Adherence at 3 Months: Data From a Randomized Trial

**DOI:** 10.2196/50242

**Published:** 2024-03-14

**Authors:** Cheryl D Stults, Kathleen M Mazor, Michael Cheung, Bernice Ruo, Martina Li, Amanda Walker, Cassandra Saphirak, Florin Vaida, Sonal Singh, Kimberly A Fisher, Rebecca Rosen, Robert Yood, Lawrence Garber, Christopher Longhurst, Gene Kallenberg, Edward Yu, Albert Chan, Marlene Millen, Ming Tai-Seale

**Affiliations:** 1 Palo Alto Medical Foundation Research Institute Center for Health Systems Research Sutter Health Palo Alto, CA United States; 2 Department of Medicine UMass Chan Medical School Worcester, MA United States; 3 Herbert Wertheim School of Public Health and Human Longevity Science University of California San Diego La Jolla, CA United States; 4 Department of Medicine University of California San Diego La Jolla, CA United States; 5 Department of Family Medicine University of California San Diego La Jolla, CA United States; 6 Research Department Reliant Medical Group Worcester, MA United States; 7 Division of Biomedical Informatics Department of Medicine University of California San Diego La Jolla, CA United States; 8 Department of Family Medicine Palo Alto Medical Foundation Sutter Health Mountain View, CA United States; 9 Digital Team Sutter Health Sacramento, CA United States; 10 Department of Medicine Division of Biomedical Informatics Research Stanford University Stanford, CA United States

**Keywords:** primary care, survey, patient adherence, adherence, self-reported, surveys, content analysis, RCT, randomized, controlled trial, controlled trials, plan, plans, willingness, experience, experiences, attitude, attitudes, opinion, opinion, perception, perceptions, perspective, perspectives

## Abstract

**Background:**

Effective primary care necessitates follow-up actions by the patient beyond the visit. Prior research suggests room for improvement in patient adherence.

**Objective:**

This study sought to understand patients’ views on their primary care visits, the plans generated therein, and their self-reported adherence after 3 months.

**Methods:**

As part of a large multisite cluster randomized pragmatic trial in 3 health care organizations, patients completed 2 surveys—the first within 7 days after the index primary care visit and another 3 months later. For this analysis of secondary outcomes, we combined the results across all study participants to understand patient adherence to care plans. We recorded patient characteristics and survey responses. Cross-tabulation and chi-square statistics were used to examine bivariate associations, adjusting for multiple comparisons when appropriate. We used multivariable logistic regression to assess how patients’ intention to follow, agreement, and understanding of their plans impacted their plan adherence, allowing for differences in individual characteristics. Qualitative content analysis was conducted to characterize the patient’s self-reported plans and reasons for adhering (or not) to the plan 3 months later.

**Results:**

Of 2555 patients, most selected the top box option (9=definitely agree) that they felt they had a clear plan (n=2011, 78%), agreed with the plan (n=2049, 80%), and intended to follow the plan (n=2108, 83%) discussed with their provider at the primary care visit. The most common elements of the plans reported included reference to exercise (n=359, 14.1%), testing (laboratory, imaging, etc; n=328, 12.8%), diet (n=296, 11.6%), and initiation or adjustment of medications; (n=284, 11.1%). Patients who strongly agreed that they had a clear plan, agreed with the plan, and intended to follow the plan were all more likely to report plan completion 3 months later (*P*<.001) than those providing less positive ratings. Patients who reported plans related to following up with the primary care provider (*P*=.008) to initiate or adjust medications (*P*≤.001) and to have a specialist visit were more likely to report that they had completely followed the plan (*P*=.003). Adjusting for demographic variables, patients who indicated intent to follow their plan were more likely to follow-through 3 months later (*P*<.001). Patients’ reasons for completely following the plan were mainly that the plan was clear (n=1114, 69.5%), consistent with what mattered (n=1060, 66.1%), and they were determined to carry through with the plan (n=887, 53.3%). The most common reasons for *not* following the plan were lack of time (n=217, 22.8%), having decided to try a different approach (n=105, 11%), and the COVID-19 pandemic impacted the plan (n=105, 11%).

**Conclusions:**

Patients’ initial assessment of their plan as clear, their agreement with the plan, and their initial willingness to follow the plan were all strongly related to their self-reported completion of the plan 3 months later. Patients whose plans involved lifestyle changes were less likely to report that they had “completely” followed their plan.

**Trial Registration:**

ClinicalTrials.gov NCT03385512; https://clinicaltrials.gov/study/NCT03385512

**International Registered Report Identifier (IRRID):**

RR2-10.2196/30431

## Introduction

Primary care is an essential component of health care in the United States, where primary care providers (PCPs) provide comprehensive and longitudinal care to patients [1]. The role of the PCP has expanded over time, with PCPs providing more diagnoses, more treatments, and more preventive services in recent years [2]. In 2015, about 25% of PCP visits were for preventive care, about 30% care for chronic conditions, and just under 40% for acute care [3]. In many instances, primary care visits generate a plan for follow-up actions that extend beyond the encounter, as care often involves tasks that the patient (and sometimes the PCP) will do in the subsequent days and weeks. Ideally, the patient and the PCP jointly decide upon and agree to a plan [4], which might involve initiating or adjusting medications, monitoring symptoms, scheduling tests, implementing lifestyle modifications, or a multitude of other steps. Adherence to plans is affected by a broad range of factors including the provider-patient relationship [5]. One meta-analysis reported a 19% greater risk of nonadherence among patients who reported their physician communicated poorly during the visit [6].

While there is a substantial body of research on patient adherence, much of it is focused on adherence in the context of specific diseases, such as diabetes [7], coronary heart disease [8], asthma [9], and depression [10]. Medication adherence has been particularly well studied; 1 meta-analysis of 50 years of adherence research found that 63% of the studies focused on medication adherence, with many fewer studies examining adherence to recommendations for changes in diet (4.8%) and exercise (2.5%) [11]. In general, overall adherence rates have been estimated to range between 50% and 75%, suggesting substantial room for improvement [11,12]. Understanding patients’ perspectives on the plan that they have (or have not) understood and agreed to and their reasons for following or not following the plan could provide insights that would help PCPs promote better adherence. To our knowledge, there have been no large, multisite studies in the United States describing patients’ perceptions of the plans that result from their primary care visits, the extent to which patients follow these plans, and their reasons for following or not.

The purpose of this study was to describe patients’ perceptions of the plans generated during primary care visits and characterize these plans. We also sought to describe patients’ reports of whether they adhered to the plan, the extent to which follow-through was related to their initial perceptions of the plan, and their reasons for adherence (or lack of adherence) to the plan.

## Methods

### Overview

The data for this analysis were collected in the context of a large multisite cluster randomized controlled trial (ClinicalTrials.gov NCT123456) evaluating the impact of 3 approaches to facilitating communication and shared decision-making in primary care encounters. The 3 approaches were 1. in-person coaching for clinicians along with patients receiving a pre-visit questionnaire in advance of their visit regarding what they wanted to talk about and a video about how to prepare for their visit; 2. Mobile app coaching for clinicians and the same 2 components for patients (pre-visit questionnaire and video), and 3. Poster in exam room to encourage shared decision making. The primary study outcomes were patient-reported perceptions of communication and decision making during the appointment. While not one of the primary outcomes, this analysis was of secondary outcomes that were a part of the original research questions to examine patient plans and how patients were then able to carry them out. A detailed description of the methods and the findings of the trial are available elsewhere [13]; we briefly summarize the relevant methods here. Because the main analysis did not detect a statistically significant difference between the 3 study arms, we do not present the findings about plans by study arm. Additionally, when coding the data, we did not see any major differences across the arms and so we are examining data across all study participants.

English-speaking adult patients with a web-based patient portal account were invited to participate in the study at least 3 days in advance of scheduled appointments with participating PCPs at each site. We selected English-speaking primary care patients as that was the group studied in the initial pilot [14]. We also selected patients with a web-based patient portal account because 2 key components of the intervention (the single-item previsit questionnaire and video) were delivered via the portal. Details about the recruitment processes, which include patient portal and email invitations, have been described elsewhere [15]. Patients who provided informed consent and completed a web-based postvisit survey within 7 days of the visit were sent a follow-up survey approximately 3 months later (also on the web). Nonrespondents to the follow-up survey were sent 2 reminder emails. Patients received a US $20 incentive for completing each survey. Survey data were collected and managed using REDCap (Research electronic data capture; Vanderbilt University) tools [16,17]. Data collection occurred from September 2019 through November 2021.

The analytic sample for the study reported here includes all patients who completed the initial postvisit survey within 7 days of the visit and also completed a 3-month survey. We focus specifically on the questions related to development and adherence to plans generated during the visit. In the initial postvisit survey, patients’ perceptions of the plan discussed during their medical appointment were assessed with 3 statements, each rated on a 10-point scale (from 0 to 9, with 0 as “strongly disagree” and 9 as “strongly agree”): (1) My doctor and I have a clear plan for what to do next about my health issues; (2) I agree with the plan my doctor and I have for taking care of my health issues; and (3) I will follow the plan my doctor and I made for taking care of my health issues. Patients were asked to enter a free-text response to the open-ended question: “Thinking about the plan that you and your doctor made, what is the most important thing that you will do over the next three months?”

In the 3-month survey, patients’ descriptions of their plan from the postvisit survey (ie, response to “the most important thing you will do”) were populated into the survey to remind patients of what they had written originally. Patients were then asked: “To what extent have you carried out the plan?” with response options of “Completely,” “Somewhat,” “Not At All,” and “This Doesn’t Apply To Me/I Didn’t Have a Plan.” Patients who responded “Somewhat” or “Not At All” were asked to select from a list of reasons for not carrying out the plan and those who responded “Completely” were asked to select what helped them adhere to the plan. Patients were able to select multiple reason options or write in a text response.

### Ethical Considerations

The study was conducted at 3 health care organizations in 2 states (California and Massachusetts). The overall study, including these secondary outcomes, was reviewed and approved by institutional review boards at Sutter Health (2017.094EXP), the University of California San Diego (#180310), and the UMass Chan Medical School (H0001310).

### Analysis

We computed means and frequencies to describe the patient characteristics and responses to the survey questions described above. We dichotomized patients’ responses to each of the 3 questions about communication into the “top box,” indicating whether the patient gave the highest score possible, that is, 9 or less than top box. This approach is often used operationally [14]. Cross-tabulation and chi-square statistics were used to examine the bivariate associations between patients’ initial postvisit ratings of their plan and reported plan adherence approximately 3 months later. We also examined the association between the type of plan and plan adherence. We also examined the association between the type of plan and plan adherence. Significance values were adjusted by the Bonferroni corrections for multiple comparisons for the various types of plans. The binary outcome of carrying out the plan was analyzed using a multipredictor logistic regression, including the 3 questionnaire responses mentioned above and adjusted for the demographic factors to demonstrate how the patients’ understanding or agreement or intention to follow the plan impacts the adherence to the plan, allowing for differences due to various characteristics. We used qualitative content analysis to categorize responses to the open-ended questions “Thinking about the plan that you and your doctor made, what is the most important thing that you will do over the next three months?” in the postvisit survey and the free-text responses to other reasons for not adhering to a plan and adhering to a plan in the 3-month survey.

We developed a codebook for the content analysis using an iterative process. Investigators and staff at each site first reviewed the free-text responses for their site to create inductive codes capturing the content evident in the responses. We then compared the codes generated at each site to determine common codes and established a formal codebook that coders at each site applied to their data. Coders flagged any text that was difficult to code and brought these responses to the full coding team, which discussed the text and determined whether modifications to the coding scheme were needed. The full coding team also adjudicated final code assignments for any difficult or questionable responses.

## Results

We present the results combining all 3 sites as we found that they were very similar during the qualitative analysis. The analytic sample for this study included 2555 patients who completed both the postvisit and 3-month surveys (3847 total completed surveys, 66.4%). Patient participants were on average 52 (SD 16.4) years old. The majority were female (n=1662, 65%), White (n=2097, 82%), non-Hispanic (n=2304, 90%), and had a 4-year college degree or higher (n=1151, 72%). Many (n=1547, 60.5%) had their visit during the COVID-19 pandemic (eg, after March 16, 2020; Table 1).

Overall, a large majority of patients selected the top box option (9=definitely agree) to indicate that they felt they had a clear plan (n=2011, 78%), agreed with the plan (n=2049, 80%), and intended to follow the plan (n=2108, 83%) discussed with their PCP.

The types of plans patients reported are summarized in Table 1. Patients could report more than 1 type of plan or a plan that included multiple components. The most common elements of the plans reported included reference to exercise (n=359, 14.1%), testing (laboratory, imaging, etc; n=328, 12.8%), diet (n=296, 11.6%), and initiation or adjustment of medications (n=284, 11.1%). The correspondence between patients’ views of their plan as reported soon after the visit and their report of whether they had followed the plan 3 months later is presented in Table 2. Patients who reported that they had a clear plan, agreed with the plan, and intended to follow the plan were all more likely to report completion of the plan 3 months later compared to those who provided less positive ratings on these items initially (*P*<.001). Table 2 also shows the percentage of patients who reported they had “Completely” followed through 3 months later for each type of plan. Patient plans related to following up with the PCP, initiating or adjusting medications, and having a specialist visit were more likely to indicate that they had completely followed the plan (*P*=.008, *P*≤.001, and *P*=.003, respectively). Patients whose plans involved lifestyle changes such as weight loss, diet, and exercise were less likely to report that they had “Completely” followed their plan than those whose plans did not involve lifestyle changes (*P*<.001).

For the multivariable logistic regression, compared to those who responded other than “definitely agree,” those patients who indicated “definitely agree” that they would follow the plan were more likely to report 3 months later that they completely followed the plan (adjusted odds ratio 1.95, 95% CI 1.48-2.58; Table 3).

Patients were able to report more than 1 reason for following or not following the plan. Patients’ reasons for completely following the plan included that the plan was clear (n=1114, 69.5%), consistent with what mattered (n=1060, 66.1%), they were determined to carry through with the plan (n=887, 53.3%), and had the support needed to carry through the plan (n=570, 33.7%; Table 4). The most common reasons for *not* following the plan were lack of time (n=217, 22.8%), decided to try a different approach (n=105, 11%), the COVID-19 pandemic impacted the plan (n=105, 11%), the plan did not fit the lifestyle (n=93, 9.7%), and the plan was no longer needed or relevant (n=90, 9.5%; Table 4).

**Table 1:** Sample characteristics (N=2555).

Characteristic		Overall sample
**Age (years)**		
	Median (IQR)	54.0 (39.0-66.0)
	mean (SD)	52.6 (16.4)
**Gender, n (%)**		
	Female	1662 (65)
	Male	838 (32.8)
	Other or missing	55 (2.15)
**Race, n (%)**		
	American Indian or Alaska Native	12 (0.5)
	Asian	266 (10.4)
	Black or African American	50 (2)
	Native Hawaiian or other Pacific Islander	11 (0.4)
	White	2097 (82.1)
	More than 1 race	68 (2.7)
	Missing	51 (2)
**Ethnicity, n (%)**		
	Hispanic	231 (9)
	Non-Hispanic	2304 (90.2)
	Missing	20 (0.8)
**Education, n (%)**		
	High school graduate or general educational diploma or less	161 (6.3)
	Some college or 2-year degree	548 (21.4)
	4-year college graduate	695 (27.2)
	More than a 4-year college degree	1151 (45)
**Index visit on or after March 16, 2020 (COVID-19 pandemic), n (%)**		
	No	986 (38.6)
	Yes	1547 (60.5)
	Missing	22 (0.9)
**Health system, n (%)**		
	A	1240 (48.5)
	B	857 (33.5)
	C	458 (17.9)
**How confident are you in filling out forms by yourself?, n (%)**		
	Extremely	2309 (90.4)
	Quite a bit or somewhat or a little bit	237 (9.3)
	Not at all	7 (0.3)
	Missing	2 (0.1)
**Type of plan, n (%)^a^**		
	Exercise	359 (14.1)
	Testing (laboratory, imaging, etc)	328 (12.8)
	Diet	296 (11.6)
	Medication management	284 (11.1)
	Specialist referral	246 (9.6)
	Monitor or control condition	218 (8.5)
	Follow-up with PCP^b^	181 (7.1)
	Lose weight	96 (3.8)
	Other strategies not specified above^c^	230 (9)
	Other preventive behaviors not specified above^d^	168 (6.6)
	Did not have a plan	17 (0.7)
**My doctor and I have a clear plan for what to do next about my health issues, n (%)**		
	Definitely agree (“top box”)	2011 (78.7)
	Less than definitely agree	544 (21.3)
**I agree with the plan my doctor and I have for taking care of my health issues, n (%)**		
	Definitely agree (“top box”)	2049 (80.2)
	Less than definitely agree	506 (19.8)
**I will follow the plan my doctor and I made for taking care of my health issues, n (%)**		
	Definitely agree (“top box”)	2108 (82.5)
	Less than definitely agree	447 (17.5)
**To what extent have you carried out the plan?, n (%)**		
	Completely	1603 (62.7)
	Not at all or somewhat	952 (37.3)

^a^Plans could include more than 1 component; categories are not mutually exclusive.

^b^PCP: primary care provider.

^c^Other strategies included physical therapist, occupational therapist, health educator, mental health therapy, herbal supplements, managing stress, and independent learning.

^d^Smoking cessation, vaccination, reducing alcohol consumption, and general comments about healthy lifestyle.

**Table 2:** Extent of the followed plan by intention and types of plan.

Characteristics		To what extent have you carried out the plan?		Odds ratio (95% CI)	Overall *P* value	
		Completely (n=1603, 62.7%), n (%)	Less than completely (n=952, 37.3%), n (%)			
**My doctor and I have a clear plan for what to do next about my health issues**						<.001
	Definitely agree (“top box”)	1323 (65.8)	688 (34.2)	1.81 (1.5-2.2)		
	Less than definitely agree	280 (51.5)	264 (48.5)	N/A^a^		
**I agree with the plan my doctor and I have for taking care of my health issues**						<.001
	Definitely agree (“top box”)	1350 (65.9)	699 (34.1)	1.93 (1.59-2.35)		
	Less than definitely agree	253 (50)	253 (50)	N/A		
**I will follow the plan my doctor and I made for taking care of my health issues**						<.001
	Definitely agree (“top box”)	1398 (66.3)	710 (33.7)	2.32 (1.89-2.86)		
	Less than definitely agree	205 (45.9)	242 (54.1)	N/A		
**Types of plan**						
	Exercise	174 (48.5)	185 (51.5)	0.51 (0.37-0.7)^b^	<.001^c^	
	Testing (laboratory, imaging, etc)	222 (67.7)	106 (32.3)	1.28 (0.9-1.84)^b^	.54^c^	
	Diet	149 (50.3)	147 (49.7)	0.56 (0.4-0.8)^b^	<.001^c^	
	Medication management	209 (73.6)	75 (26.4)	1.75 (1.19-2.63)^b^	<.001^c^	
	Specialist referral	181 (73.6)	65 (26.4)	1.73 (1.15-2.68)^b^	.003^c^	
	Monitor or control condition	152 (69.7)	66 (30.3)	1.4 (0.92-2.19)^b^	.31^c^	
	Follow-up with primary care provider	135 (74.6)	46 (25.4)	1.81 (1.12-3.02)^b^	.008^c^	
	Lose weight	41 (42.7)	55 (57.3)	0.43 (0.23-0.77)^b^	<.001^c^	
	Other strategies not specified above	127 (55.2)	103 (44.8)	0.71 (0.48-1.05)^b^	.16^c^	
	Other preventive behaviors not specified above	100 (59.5)	68 (40.5)	0.86 (0.55-1.38)^b^	≥.99^c^	

^a^N/A: not available.

^b^Bonferroni-corrected 99.5% CI.

^c^Bonferroni-corrected *P* value.

**Table 3:** Adjusted logistic regression of extent followed plan.

Explanatory variable			Odds ratio (95% CI)		Overall *P* value
My doctor and I have a clear plan for what to do next about my health issues: Definitely agree (“top box”; Reference: Less than definitely agree)			1.16 (0.85-1.57)		.35
I agree with the plan my doctor and I have for taking care of my health issues: Definitely agree (“top box”; Reference: Less than definitely agree)			1.15 (0.82-1.60)		.42
I will follow the plan my doctor and I made for taking care of my health issues: Definitely Agree (“top box”; Reference: Less than definitely agree)			1.95 (1.48-2.58)		<.001
Age			1.0 (1.0-1.0)		.80
Gender: non-female or missing (Reference: Female)			1.03 (0.87-1.23)		.71
Race: non-White (Reference: White)			1.10 (0.87-1.39)		.43
Education: less than a 4-year college degree (Reference: 4-year college graduate)			0.92 (0.76-1.11)		.39
Index visit on or after March 16, 2020: Yes (Reference: No)			1.05 (0.88-1.25)		.60
**Health system (Reference: A)**					
	B	0.93 (0.77-1.13)		.46	
	C	0.81 (0.64-1.02)		.07	

**Table 4:** Reasons for following the plan.

						Values, n (%)
**Patients who reported “Completely” followed the plan (n=1603)**						
	**What helped you to carry out the plan? (Select ALL that apply**)					
		The plan was clear to me			1114 (69.5)	
		The plan was consistent with what mattered most to me			1060 (66.1)	
		I was determined to carry it through			887 (55.3)	
		I had the support needed to carry it through			540 (33.7)	
		Other			19 (1)	
**Patients who reported “Not at All” or “Somewhat” followed plan (n=952)**						
	**There are many reasons why people do not carry out a plan exactly. Please select ALL reasons that apply to you**					
		**5 most frequently selected responses**				
			Lack of time	217 (22.8)		
			Try a different approach	105 (11)		
			Did not fit with my lifestyle	93 (10)		
			Plan was no longer needed or relevant	90 (9)		
			Plan was not working	39 (4)		
		**5 most frequently written in as “Other”**				
			Impact from COVID-19	105 (11)		
			Plan in progress	77 (8)		
			Life events or activities of daily living impact	44 (5)		
			Not motivated to complete the plan	40 (4.2)		
			Other health issues	33 (3)		

## Discussion

### Principal Findings and Comparison With Prior Work

In this multisite study of primary care visits, we found that the majority (roughly 80%, n=2049 and 2108, respectively) of patient participants felt that they agreed with and would follow the plan that resulted from their primary care visit. These views gathered soon after their PCP visit were statistically significantly associated with the likelihood of completely following their plan after controlling for other factors. Patients who did not feel clear about the plan or who were not in agreement with the plan were much less likely to follow the plan. This is consistent with the finding reported in 1 meta-analysis, which found that the odds of patient adherence are 2.16 times higher if a physician communicates effectively [6]. This suggests that future studies could evaluate whether providers could improve treatment adherence through “teach-back,” where they confirm patients understand and are in agreement with the plan before the end of the visit [18].

We found that many patients reported that they did not completely follow plans related to weight loss, diet improvement, and increased exercise. These findings are consistent with other studies that found patients tended to be more adherent to circumscribed treatment regimens (eg, medication use) as compared to complex health behavior change efforts such as diet [11]. Given these challenges and limited insights provided through research, patients and providers may need to be proactive and anticipate difficulties in these areas. The evidence suggests that “knowledge alone is not sufficient to enhance adherence in recommendations involving complex behavior change” [19] like modifying diet and exercise. Providers should consider simplifying proposed regimen changes to better “match patients’ activities of daily living” [19]. Motivational interviewing could be used to better help the patient identify and set their own goals and identify both potential barriers and ways to overcome them [20]. Some other potential ways to improve provider communication include additional training on how to provide empathy [21] as empathy has been shown to improve both adherence and patient satisfaction [22,23] and training on agenda setting can help the flow of the visit and improve the overall interaction [24].

Many patients in our study identified lack of time as the reason for not fully adhering to their plan, which is also consistent with previous research [25]. Patients in our study were further impacted by the COVID-19 pandemic, which caused nonessential medical appointments to be canceled, affecting patients’ plans to follow up with their PCPs or specialists, or to complete laboratory testing. Gyms were closed, which impacted some patients’ plans to exercise. Other researchers have documented the impact of COVID-19 on exercise in the general population; for instance, a survey conducted in November 2020 found that over 25% of respondents said that they still did not go out to walk, hike, or exercise even after the initial pandemic lockdown restrictions were lifted [26].

### Limitations

A major limitation of our study is our reliance on patient self-report. Participating patients may have only reported limited descriptions of their plans, whereas there may have been more in-depth discussions with their PCP about the plans and next steps during the actual conversations. We also did not provide an opportunity for patients to identify elements of their physician’s recommendations, and future research should consider potentially incorporating this aspect. We did not capture the PCP’s perspective on the encounter or the plan, and doing so would have allowed us to examine the correspondence between the patient’s understanding of the plan and the PCP’s understanding of what had been agreed to (or what was most important). Our analysis assumed that all patient plans carried equal clinical importance and we did not evaluate for complexity of the plan. These are 2 factors that could potentially impact plan adherence. Additionally, our population was predominately White and nearly half have more than a 4-year college degree; their ability to understand instructions and reasoning to carry out the plan may not be representative of what might be found in a general population. Finally, our study evaluated plan adherence after 3 months so that may be insufficient time to expect resolution of some more complex medical issues.

### Conclusions

In this multisite study of patients’ views on their primary care visits and the plans generated during these visits, we found that overall, patients’ initial assessment of their plan as clear, their agreement with the plan, and their initial willingness to follow the plan were all strongly related to their self-reported completion of the plan 3 months later.

## Abbreviations

PCP: primary care provider
REDCap: Research Electronic Data Capture
